# Community Genetics screening in a pandemic: solutions for pre-test education, informed consent, and specimen collection

**DOI:** 10.1038/s41431-022-01251-2

**Published:** 2023-01-11

**Authors:** Bronwyn Terrill, Lauren McKnight, Angela Pearce, Heather Gordon, William Lo, I-Chieh Jennifer Lee, Monica Runiewicz, Alex Palmer, Lesley Andrews, Edwin Kirk, Daniel Goldberg, John Tucker, David Murray, Warren Kaplan, Sarah Kummerfeld, Leslie Burnett

**Affiliations:** 1 Kinghorn Centre for Clinical Genomics, Garvan Institute of Medical Research, Darlinghurst, NSW 2010 Australia; 2 School of Clinical Medicine, St Vincent’s Clinical Campus, UNSW Sydney, Darlinghurst, NSW 2010 Australia; 3 NSW Health Pathology, Randwick, NSW 2031 Australia; 4 Community Genetics Program (NSW), Woollahra, NSW 2034 Australia; 5 School of Clinical Medicine, Prince of Wales Clinical Campus, UNSW Sydney, Randwick, NSW 2031 Australia; 6 Wolper Jewish Hospital, Woollahra, NSW 2034 Australia; 7 NSW Health, Prince of Wales Hospital, Randwick, NSW 2031 Australia; 8 Northern Clinical School, Faculty of Medicine and Health, University of Sydney, St Leonards, NSW 2065 Australia

**Keywords:** Population screening, Genetic testing

## Abstract

A Community Genetics carrier screening program for the Jewish community has operated on-site in high schools in Sydney (Australia) for 25 years. During 2020, in response to the COVID-19 pandemic, government-mandated social-distancing, ‘lock-down’ public health orders, and laboratory supply-chain shortages prevented the usual operation and delivery of the annual testing program. We describe development of three responses to overcome these challenges: (1) pivoting to online education sufficient to ensure informed consent for both genetic and genomic testing; (2) development of contactless telehealth with remote training and supervision for collecting genetic samples using buccal swabs; and (3) a novel patient and specimen identification ‘GeneTrustee’ protocol enabling fully identified clinical-grade specimens to be collected and DNA extracted by a research laboratory while maintaining full participant confidentiality and privacy. These telehealth strategies for education, consent, specimen collection and sample processing enabled uninterrupted delivery and operation of complex genetic testing and screening programs even amid pandemic restrictions. These tools remain available for future operation and can be adapted to other programs.

## Introduction

A Community Genetics carrier screening program for the Jewish community has operated on-site in high schools in Sydney (Australia) for 25 years. During 2020, in response to the COVID-19 pandemic, government-mandated social-distancing, “lock-down” public health orders, and laboratory supply-chain shortages prevented the usual operation and delivery of the annual testing program. Here, we describe solutions deployed to overcome COVID-19 pandemic challenges, adaptable to other genetic testing and screening programs.

## Carrier screening in the community

Genetic carrier testing or reproductive carrier screening (referred to here as “carrier screening”) [1] is a test for healthy individuals before or during pregnancy to see if they have an increased likelihood of having a child with an autosomal or X-linked condition.

Community-wide carrier screening has been offered to groups whose ancestry makes them at increased risk of being a genetic carrier of various conditions. The best-known of these are screening programs for Tay-Sachs disease (TSD), an autosomal recessive, highly-penetrant, incurable childhood condition particularly prevalent in people with Ashkenazi Jewish (AJ) ancestry.

Within Australia, there are ~120,000 AJ individuals residing mainly in Sydney and Melbourne. A Community Genetics program has operated continuously in Sydney since 1995 [2, 3]; its operation has led to the disappearance of TSD-affected births in the at-risk tested population [4]. This program is run by Sydney’s Wolper Jewish Hospital (https://www.genetics.wolper.com.au) with a primary focus of organizing annual education and pre-conception testing in five high schools in metropolitan Sydney with high proportions of enrolled Jewish students.

In this Community Genetics program, year 11 students (irrespective of ancestry or religion) attend a 1-h compulsory genetics education session with a genetics or educational professional. Several days later voluntary, follow-up testing is provided at participating high schools, where students can formally consent to collection of a saliva or buccal swab sample, and genetic carrier testing is performed in a clinically-accredited pathology laboratory. All tested students receive a written report; any student found to be a genetic carrier is also offered genetic counseling. This Community Genetics program has been extensively studied and evaluated over 25 years [2–5], showing the effectiveness of pre-test group education in achieving informed consent for carrier screening using both specific genetic tests [3] and genetic panels [5].

## Community genetic screening no longer possible during the pandemic

In March 2020, COVID-19 pandemic public health orders introduced mandated social distancing requirements. Supply-chain shortages emerged and the testing laboratory could not guarantee to extract DNA from samples collected for the program. Social distancing rules prevented non-school-staff from attending school premises to deliver education or collect specimens, exacerbated by infection risks of collecting oral samples. It became clear that the Community Genetics program’s standard testing protocol could not be supported.

## Solutions

### 1: Online genetic education linked to informed consent gateways

**Fig. 1:**
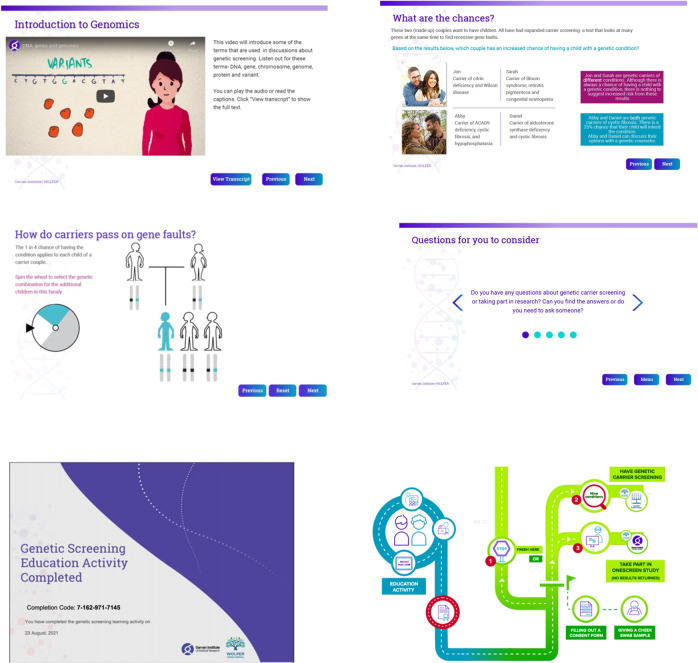
Sample screenshots of the online educational program and related resources. From top left: Educational video on basic genetic principles; potential clinical scenarios; interactive activity to illustrate inheritance models and random segregation; FAQs (Frequently Asked Questions); certificate with unique verification code to confirm completion of pre-test education program; cartoon roadmap to guide participants through personal decision-making.

The genetics education previously delivered as a group presentation was adapted as a multimedia course that could be delivered online or in-school (Fig. 1). Education resources developed included interactive media, progression tracking, and real-time feedback. Students were provided with unique, anonymised codes, permitting them to access this material remotely, revisit content and share information with family. Education development and evaluation are described in the Supplementary Material (Supplementary S1).

The education was intended to engage students and increase their genetic carrier screening knowledge, to facilitate consideration of the benefits, risks, and implications of screening, and to enable students’ decision-making about genetic screening and genomics research. Education sessions were delivered in five high schools during 2020. As public health orders shifted, delivery modes varied according to school preferences. Module efficacy was evaluated with surveys administered immediately before and after the education session.

A unique digital certificate was generated and issued to students on completion of the education module; this was verified prior to proceeding to testing. For students not wishing to participate in testing, information was provided for how they could access at any future time a community outreach clinic, or a genetics clinic in the State healthcare system.

### 2: Contactless testing protocols using telehealth and buccal swab samples

Approximately 1 week post-education, all students were offered the opportunity to undertake genetic carrier testing through on-site collection at each school. Depending on prevailing COVID-19 restrictions, students were offered either standard enrollment and self-collection of buccal swab specimens supervised by on-site, socially-distanced collection staff, or an alternative procedure involving no in-person supervision, where students enrolled and self-collected their specimen through a telehealth video link over an iPad with remotely-located collection staff. A YouTube video (https://www.youtube.com/watch?v=SnMZpSS4K18) demonstrated correct collection technique, and this video was also embedded in the online learning materials.

### 3: GeneTrustee protocol and database technology to maintain clinically-accredited testing standards (see Supplementary Materials and Fig. 2)

**Fig. 2:**
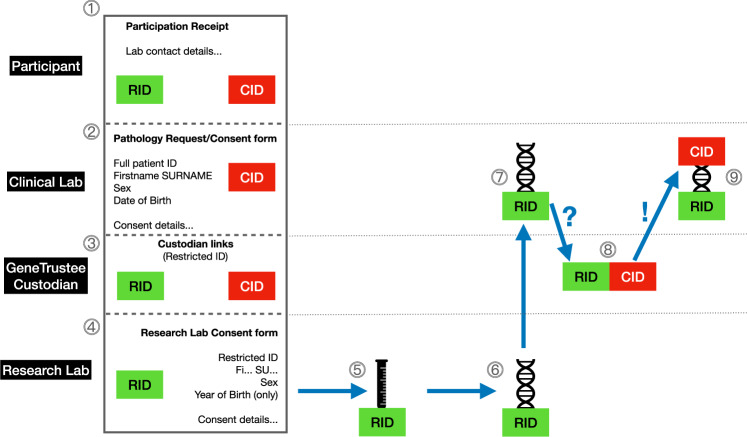
The GeneTrustee [7] multi-part request/consent form and workflow. The form is organized into four sections, each easily separated using pre-perforated paper. The top section  is the “receipt” and is given to the participating student. It contains contact details for all laboratories, and two identification numbers: the clinical laboratory identification (CID) and the research laboratory identification (RID). The second section  is for the clinical laboratory, and contains full patient identifiers, [6] consent for testing and the CID. The third section  is for the Custodian of the GeneTrustee, and it contains both the CID and RID. The fourth section  is for the research laboratory, and it contains only restricted identifiers (in this study, we used the first two letters only of first and last names, sex, and year-only of birth), sufficient to uniquely identify the case, but insufficient to reveal personally identifiable information, plus consent for research, and the RID. The sample is collected and labeled only with the RID , and the research laboratory extracts DNA  and forwards it to the clinical laboratory . In step , the clinical laboratory contacts the Custodian of the GeneTrustee, providing the RID and (without revealing the patient’s personal identifiers) additional restricted identifiers taken from the full clinical identifiers); the GeneTrustee matches this information and returns the corresponding CID to the clinical laboratory , which now has a fully re-identified DNA sample from the patient’s sample, obtained without the research laboratory or the Custodian ever having known or been provided with the identity of the patient.

To reduce pressure on the clinical laboratory, DNA samples were collected and extracted by a separate research laboratory. The research laboratory was not permitted to record the personal identification data of any participant. However, as this was a genetic pathology test, the clinical diagnostics laboratory was required to identify the specimen using a minimum of two, and ideally three, primary patient identifiers [6]. To overcome these two seemingly incompatible requirements, we adapted the GeneTrustee protocol [7], by developing a multi-part request/consent form (Fig. 2). The clinical laboratory received a section containing personally identifiable information while the research laboratory received a different section, containing only a minimum dataset of three abbreviated primary identifiers [6], sufficient to identify the accession, but insufficient to identify the participant. Additionally, a unique accession identification number was applied to each sample (see Fig. 2). The sample was processed by the research laboratory, which extracted DNA for the clinical laboratory. Finally, the clinical laboratory was able to match the (deidentified) DNA extract from the research laboratory, with the appropriate clinical identifiers, by contacting the Custodian of the GeneTrustee, who was the only party able to relink these items, even though they did not know the identity of the participants (Fig. 2).

## Resumption of community genetic screening in a pandemic

Up to 300 students completed the education, with 250 consenting to participate in the subsequent carrier testing sample collection. Of these 250 participants, two declined involvement of the research laboratory and were directly collected by the clinical laboratory. After the education module, both carrier screening knowledge scores and attitudes toward carrier screening increased (Supplementary Fig. S1).

Of the 248 samples collected using the GeneTrustee protocol using buccal swabs, all contained sufficient DNA for genetic testing. However, two samples were subsequently recollected, as these two students had a combination of names, sex and year of birth that were insufficiently dissimilar using the abbreviated demographics. Although their accession numbers were different, using a full date-of-birth, or adding a random number (analogously to a cryptographic salt [8]) to each accession could have avoided this problem.

No students attempted to copy, duplicate, or forge the certificate to access testing without completing the education: all certificates presented at sample collection contained valid unique certificate numbers.

## Reflections on screening in a pandemic

In the face of a pandemic, with constantly changing public health orders and disruption of clinical laboratory supply chains, we continued uninterrupted provision of genetic screening at scale. We used a combination of: flexible online education; buccal swabs for DNA testing collected through contactless and remote telehealth supervision; and a novel GeneTrustee identification protocol.

In this local scenario of community genetic screening, pivoting to online education programs enables wider access to genetic testing programs beyond those currently offered using traditional in-person protocols in limited numbers of community high schools. It offers potential to provide testing education in a broader geographic region and in schools where there are insufficient numbers of at-risk students to justify an on-site program. However, on a broader scale, this process is also readily adaptable to genetic testing of anyone whose access to laboratories is limited by geography or personal circumstances, with telehealth clinician support.

Completion certificates generated by the education modules provide assurance that patients or participants have completed education prior to screening. The use of self-collection buccal swabs [9] with online video instructions and remote telehealth supervision options, reduces the need for on-site specimen collection staff, especially for smaller sites with few participants. The GeneTrustee protocol enabled a non-clinical laboratory to collect and process specimens without any personally identifiable clinical information, and yet met all clinical accreditation requirements [6] for chain-of-custody identification in healthcare.

The need to keep a genetic screening program functional throughout the COVID-19 crisis has resulted not only in tools that can improve schools-based testing but also a model that may be utilized to enable broader genetic screening at scale. Our open-source education resources, tools and workflows are relevant to all genetic screening programs and management of any individual or patient cohort. Although we used the GeneTrustee protocol only for specimen collection and DNA extraction, it is a complete framework that includes the subsequent processing and secure storage of genomic information.

These strategies for education, consent, specimen collection and sample processing enabled uninterrupted delivery and operation of complex genetic testing and screening programs even amid pandemic restrictions. These tools remain available for future operation and scaling of expanded screening protocols and can be adapted to other programs.

## Supplementary information

Supplementary Materials

## Data Availability

The datasets generated and/or analyzed during the current study are available from the corresponding author on reasonable request.
