# Supplementary material for: Community Genetics screening in a pandemic: solutions for pre-test education, informed consent, and specimen collection
Source: Eur J Hum Genet. 2023 Jan 11;31(3):257–61. doi: 10.1038/s41431-022-01251-2 (PMC9832404; doi:10.1038/s41431-022-01251-2)
Supplement: Supplementary file 1 — Supplementary Materials [file 41431_2022_1251_MOESM1_ESM.docx]

**SUPPLEMENTARY MATERIALS**

Terrill, B., McKnight, L., Pearce, A., Gordon, H., Lo, W., I-Chieh, J.L., Runiewicz, M., Palmer, A., Andrews, L., Kirk, E., Goldberg, D., Tucker, J., Murray, D., Kaplan, W., Kummerfeld, S., Burnett, L. (2022). Community Genetics screening in a pandemic: Novel solutions for pre-test education, informed consent, and specimen collection

**CORRESPONDING AUTHOR**

Prof. Leslie Burnett, [leslie.burnett@garvan.org.au](mailto:leslie.burnett@garvan.org.au)

**GENETICS EDUCATION**

**Detailed Methods**

*Education development.* The education was developed consistent with the high school science syllabus, previously validated education approaches^1–3^ and best practice online learning approaches^4^ to increase relevance and conceptual understanding. Instructional designers referenced contemporary genetic literacy frameworks^5^ together with frameworks for informed choice Multidimensional Model of Informed Consent, underpinned by Azjen’s Theory of Planned Behaviour^6^. The education was iteratively reviewed by genetic health professionals and by members of community genetics and youth panels, aged 16-20, covering topics such as the nature of genetics and heredity, Mendelian (particularly autosomal recessive) inheritance (see **Supplementary table S1**). It also included examples of common inherited conditions in various populations (including TSD in the Ashkenazi Jewish community). Information resources highlighting key statistics, testing facts, genetic risk, and frequently asked questions were also provided for the students’ parents.

**Supplementary Table 1: Education outline**

| **Module section** | **Description** |
| --- | --- |
| Genes and genetic conditions | Videos and interactive information about genetic conditions and their causes, including what genes are; how genes can affect health; how genetic conditions can be inherited in families; and the differences in frequency of genetic conditions in certain populations. |
| Genetic carrier screening | Definitions of genetic screening, how genetic tests are performed and the options for carrier couples. This section also included personal stories from parents and young adults who have been affected by genetic conditions. |
| Genomics across a lifetime | Exploration of the differences between genetic and genomic screening and the potential uses of genomic information. |
| Making decisions | An introduction to ideas about what makes a ‘good’ decision and opportunities to weigh up risks and benefits, and to consider options. |
| Summary:  ‘Take-home messages’ | A summary of what genes are, how they cause genetic conditions, and how carrier screening works, together with a reminder of the decisions that students could make. This section also included a short video demonstrating the cheek swab collection procedure. |

*Education evaluation.* Questionnaires included measures of student knowledge (both pre- and post-education), attitudes (pre- and post-education), evaluation of the module (post-education only) and intent to participate in screening or research (post-education only), as facets of informed choice^7^ adapted from previous studies.^1–3,8^ Effects on students’ genetic carrier screening knowledge, attitudes and intent (items listed in **Supplementary Table S2**) are reported in this paper.

**Knowledge** items answered correctly were scored 1 and incorrect responses were scored as zero. **Attitude and intent** responses were scored as frequencies and an attitude score of four was created as in previous studies^1–3^ based on binary variables, where Agree = 1; Disagree/Unsure = 0.

**Supplementary table 2: Items included in student knowledge, attitude, intent and engagement analyses to evaluate the education module.**

| **Item** | **Variables** |
| --- | --- |
| **Knowledge of genetic carrier screening** |  |
| Everyone is born carrying a number of faulty genes in the cells of their body | True/ False/Unsure |
| A carrier has one copy of an autosomal recessive gene fault | True/ False/ Unsure |
| Carriers of a recessive condition will develop the condition at some time in their life | True/ False/ Unsure |
| Some faulty genes are more common in people with particular ancestry | True/ False/ Unsure |
| A person can be a carrier of faulty genes for more than one genetic condition | True/ False/ Unsure |
| A baby can inherit an autosomal recessive condition if only one parent is a carrier | True/ False/ Unsure |
| If a couple has a child with a recessive genetic condition, it is unlikely that their next child will have the same condition | True/ False/ Unsure |
| Disorders such as Tay Sachs disease and cystic fibrosis can be caught from affected individuals | True/ False/ Unsure |
| Tay Sachs disease only affects people with Ashkenazi Jewish heritage | True/ False/ Unsure |
| A negative result from a genetic carrier test means you cannot have a child with that condition | True/ False/ Unsure |
| **Attitude to genetic carrier screening** |  |
| Genetic carrier screening is the right thing to do | Agree / Unsure / Disagree |
| High school is a good time to offer genetic carrier screening | Agree / Unsure / Disagree |
| If both partners in a couple carry the faulty gene for the same condition, testing should be offered during pregnancy | Agree / Unsure / Disagree |
| Everyone should be able to have genetic carrier screening for every condition that is available even if the test is not relevant to them based on their ancestry | Agree / Unsure / Disagree |
| If you found out that you were a carrier for a faulty gene that may  cause a particular condition in your children, how would you feel? | |
| I think that I would talk to my partner about my result before considering children | Agree / Unsure / Disagree |
| I think that I would feel worried about my own health | Agree / Unsure / Disagree |
| I think that I would feel scared | Agree / Unsure / Disagree |
| think that I would feel happy with my decision to be tested | Agree / Unsure / Disagree |
| **Intent to participate in genetic carrier screening** |  |
| I plan to have carrier testing for conditions that are common to people of my ancestry within the next few months | Agree / Unsure / Disagree |
| **Engagement (post-questionnaire only)** |  |
| The information in this module was relevant to me | Agree / Unsure / Disagree |
| The information in this module was easy to understand | Agree / Unsure / Disagree |
| This module was easy to use | Agree / Unsure / Disagree |
| I feel that this module supported me to make decisions about carrier testing and genomic screening | Agree / Unsure / Disagree |
| Decisions about carrier testing or genomic screening would be hard for me to make | Agree / Unsure / Disagree |
| I feel like I know the benefits of carrier testing and genomic screening | Agree / Unsure / Disagree |
| I feel like I know the risks of carrier testing and genomic screening | Agree / Unsure / Disagree |
| I feel like I could choose whether participating in genomic research or genetic screening is right for me | Agree / Unsure / Disagree |

Paired-sample t-tests were conducted to assess differences across the two time points (pre- and post-) and *post-hoc* power calculations indicated β >0.80; the majority were in the range of 0.99 - 1.00. Pairwise deletion was used for all analyses. Frequencies and mean scores were calculated for the engagement evaluation items (post-education session only). For the Engagement/evaluation responses, items were scored as:

Agree = 3, Unsure = 2, Disagree = 1; higher mean scores indicate more agreement.

**Results**

Of 225 students who commenced the questionnaires, an evaluation of the educational effectiveness of this online program is reported for 190 students who completed both knowledge and attitude questions on the pre- and post-questionnaires; 185 students completed the intent question. Students were excluded due to duplicate surveys, missing sections, meaningless or response-set answers, or if they did not consent to data use for research.

Carrier screening knowledge scores were significantly higher immediately following the education session compared to immediately before (**Fig. 3(a)**) [Scores could range from 0 to 10. *t*(189) = 8.02, *p* <. 001, Mean Difference = 1.44, 95% CI = 1.06-1.76]^2^.   The effect size was medium (*d* = 0.60) and β = 1.00.  Low-scoring questions (answered correctly by fewer than 60% of students in the post-session questionnaire) were “A baby can inherit an autosomal recessive condition if only one parent is a carrier” (54.2% correctly answered ‘false’), and “A negative result from a genetic carrier test means you cannot have a child with that condition” (56.3% correctly answered ‘false’).

**Supplementary Figure 1: Summary of survey findings.** a) Knowledge scores. Boxplots with Tukey fences for score out of 10 questions related to carrier screening, before and after education session. b) Attitudes to carrier testing. Proportion of students agreeing with attitude statements before and after education session. c) Evaluation of education session. Responses given to statements about the module (asked only after the education session).

Attitudes towards carrier screening were positive prior to education and increased further post-education (**Fig. 3(b)**). From core attitude questions used in prior studies to generate an attitude score out of four, there was a small but significant difference in mean attitude score before (3.35) and after (3.63) education [*t*(189) = 4.00, *p* <. 001, Mean Difference = 0.27, 95% CI = 0.14-0.41]. The effect size was small (*d* = 0.30) and β = 0.99.

Post-education evaluation included relevancy, use, understandability and decision-making support. Most students agreed that the information was relevant to them, was easy to use and understand and that the module supported them to make decisions about carrier testing and genomic screening. Evaluation scores were slightly higher in schools that received a face-to-face presentation, but differences in delivery (including length of session and engagement context) prevent drawing of clear conclusions. However, engagement was high, and knowledge and attitude scores increased at each school.

Questionnaires evaluating the education session included a subset of questions used in previous studies.^1–3^ As noted by Barlow-Stewart *et al*.^2^, baseline (pre-education) knowledge and attitude scores were high in this community yet increased further following the education sessions. The lowest scoring questions continue to be those relating to the challenging concept of residual risk. This includes questions about whether a child can inherit a recessive condition if “only one parent is a carrier” or following “a negative result from a genetic carrier test”. Communication of uncertainty and the limitations of screening technologies is a subject of ongoing research.^9^

At the conclusion of the module, 90% of students felt they knew the benefits of testing and screening, with slightly fewer (79%) indicating they knew the risks. Regarding decision-making, 30.5% (n=58) agreed that decisions about carrier testing or genomic screening would be hard to make. However, most students (92%, *n* = 188) agreed with the statement “I feel like I could choose whether participating in genomic research or genetic screening is right for me”. At that point in time, following the education session, more than 4 in 5 (82%, n=151) intended to have carrier screening, 14% were unsure, and the remaining 4% did not intend to participate.

**COLLECTION AND TESTING**

**Consent for testing.** Within the State of New South Wales in Australia, the age of consent for medical procedures is 16 years, and additionally, consent can be given by a “mature minor” for medical procedures at even younger ages^10^. In keeping with the Community Genetics Program’s past practice, we adopted 16 years as the minimum age for students consenting to participate in testing; for students younger than 16 on the day of sample collection, we required both the student’s consent as well as written consent of at least one of the student’s parents or legal guardians.

**Extraction and clinical laboratory testing.**

DNA was extracted by a research laboratory (Kinghorn Centre for Clinical Genomics, NATA/RCPA accreditation number 19887) accredited for sample collection and DNA extraction, using QIAamp DNA Blood Mini Kit on the QIAcube connect ([www.qiagen.com](http://www.qiagen.com)). Validation studies confirmed extracted DNA was of sufficient yield and quality for targeted DNA sequencing methodologies implemented by the clinical laboratory.

Deidentified DNA extracts were received from the first (research) laboratory by a second (clinical diagnostics) laboratory (NSW Health Pathology, Randwick campus, NATA/RCPA accreditation number 2206) accredited for genetic carrier testing. The clinical diagnostics laboratory reidentified the specimens by approaching the Custodian of the GeneTrustee (**Fig. 2**).

**GENE TRUSTEE**

Research labels and clinical labels were attached to the relevant sections of the multi-part GeneTrustee form (**Fig. 2**), and the different sections of the form were then separated from each other. In this way, the clinical laboratory received fully-identified pathology request forms (but no sample) labelled with a Clinical Identifier (CID), the research laboratory received de-identified samples and a de-identified request form labelled with a Research Identifier (RID), while the Custodian of the GeneTrustee received matched pairs of (CID and RID) codes with truncated student names and student year of birth, but no other personal identifiers. After DNA extraction of all samples, the research laboratory provided DNA extracts (labelled with the RID) to the clinical laboratory. The clinical laboratory then contacted the Custodian of the GeneTrustee, who advised which was the CID for each corresponding RID, and from this, the clinical laboratory was able to match the correct specimen with the corresponding full set of clinical identifiers.

**REFERENCE LIST (FOR SUPPLEMENTARY MATERIALS)**

1 Barlow-Stewart K, Burnett L, Proos AL *et al.* A genetic screening programme for Tay-Sachs disease and cystic fibrosis for Australian Jewish high school students. *J Med Genet* 2003; 40: e45.

2 Barlow-Stewart K, Bardsley K, Elan E *et al.* Evaluating the model of offering expanded genetic carrier screening to high school students within the Sydney Jewish community. *J Community Genet* 2021. doi:10.1007/s12687-021-00567-8.

3 Gason AA, Metcalfe SA, Delatycki MB *et al.* Tay Sachs disease carrier screening in schools: Educational alternatives and cheekbrush sampling. *Genet Med* 2005; 7: 626–632.

4 Mayer RE. Using multimedia for e‐learning. *J Comput Assist Lear* 2017; 33: 403–423.

5 Boerwinkel DJ, Yarden A, Waarlo AJ. Reaching a Consensus on the Definition of Genetic Literacy that Is Required from a Twenty-First-Century Citizen. *Sci Educ-netherlands* 2017; 26: 1087–1114.

6 Ajzen I. The theory of planned behavior. *Organ Behav Hum Dec* 1991; 50: 179–211.

7 Marteau TM, Dormandy E, Michie S. A measure of informed choice. *Health Expect* 2001; 4: 99–108.

8 Langer MM, Roche MI, Brewer NT *et al.* Development and Validation of a Genomic Knowledge Scale to Advance Informed Decision-Making Research in Genomic Sequencing. *Mdm Policy Pract* 2017; 2: 2381468317692582.

9 Umstead KL, Han PKJ, Lewis KL *et al.* Perceptions of uncertainties about carrier results identified by exome sequencing in a randomized controlled trial. *Transl Behav Med* 2020; 10: 441–450.

10 Wallace M. *Health Care and the Law*. 3rd edn. Thomson Lawbook Co: Sydney, NSW, Australia, 2001.
